# PhosphoHunter: An Efficient Software Tool for Phosphopeptide Identification

**DOI:** 10.1155/2015/382869

**Published:** 2015-01-12

**Authors:** Alessandra Tiengo, Lorenzo Pasotti, Nicola Barbarini, Paolo Magni

**Affiliations:** Dipartimento di Ingegneria Industriale e dell'Informazione, Università degli Studi di Pavia, Via Ferrata 5, 27100 Pavia, Italy

## Abstract

Phosphorylation is a protein posttranslational modification. It is responsible of the activation/inactivation of disease-related pathways, thanks to its role of “molecular switch.” The study of phosphorylated proteins becomes a key point for the proteomic analyses focused on the identification of diagnostic/therapeutic targets. Liquid chromatography coupled to tandem mass spectrometry (LC-MS/MS) is the most widely used analytical approach. Although unmodified peptides are automatically identified by consolidated algorithms, phosphopeptides still require automated tools to avoid time-consuming manual interpretation. To improve phosphopeptide identification efficiency, a novel procedure was developed and implemented in a Perl/C tool called PhosphoHunter, here proposed and evaluated. It includes a preliminary heuristic step for filtering out the MS/MS spectra produced by nonphosphorylated peptides before sequence identification. A method to assess the statistical significance of identified phosphopeptides was also formulated. PhosphoHunter performance was tested on a dataset of 1500 MS/MS spectra and it was compared with two other tools: Mascot and Inspect. Comparisons demonstrated that a strong point of PhosphoHunter is sensitivity, suggesting that it is able to identify real phosphopeptides with superior performance. Performance indexes depend on a single parameter (intensity threshold) that users can tune according to the study aim. All the three tools localized >90% of phosphosites.

## 1. Background

Phosphorylation is a chemical reaction taking place in cells, in which a phosphate group (PO_4_) is added, by a kinase, to the amino acids serine, threonine, and tyrosine of a protein. It is a posttranslational modification (PTM) that affects both the biological action of the protein and the molecular weight by increasing the mass of the involved amino acids of about 80 Da. The phosphorylation mechanism plays a crucial role under both normal and pathological conditions.

The characterization of a target protein in terms of localization of the phosphorylation sites has become a topic of interest in proteomics, fostering the development of many analytical approaches [1–3]. Tandem mass spectrometry (MS/MS) coupled with liquid chromatography (LC) is the most widely used approach for such kind of studies [4, 5]. Briefly, the LC-MS/MS analysis of phosphorylated peptides includes the following:

1. enzymatic digestion of the proteins in a biological sample, to obtain a pool of shorter peptides;
2. separation and isolation of peptides from other compounds through LC, usually combined to a phosphopeptide enrichment methodology such as immobilized metal affinity chromatography (IMAC) or metal oxide affinity chromatography (MOAC);
3. MS/MS analysis of the isolated peptides;
4. evaluation of the MS/MS spectra through suitable algorithms in order to identify phosphopeptides and the localization of their phosphorylated amino acids.

MS is used to measure the mass/charge ratio (*m/z*) of charged molecules with high precision [6]. MS/MS is based on two mass analyzers. The first one allows to select, after ionization, a peptide in a given* m/z* range. Then, the selected peptide (called precursor) undergoes fragmentation via, for example, the traditional collision induced dissociation (CID) method, to form ions that are detected by the second mass analyzer [7].

The output of MS/MS is the representative mass spectrum of each analyzed precursor, that is, a list of all the* m/z* values of the detected ions generated by fragmentation of the precursor with the corresponding intensities. Therefore, the representative mass spectrum contains indirect information about the mass of the amino acids belonging to the sequence of an ionized peptide. All the detected MS/MS spectra are usually processed to filter out noise and hence to select the most relevant peaks, as proposed, for example, in [8, 9]. Finally, the resulting spectrum must be processed by means of proper algorithms to identify the amino acid sequence of the peptide.

Peptide identification algorithms are typically based on the comparison between the experimental MS/MS spectra and the so-called theoretical MS/MS spectra, obtained by applying, for each protein sequence, suitable enzymatic digestion rules (*in silico* digestion) followed by an* in silico* fragmentation of the obtained peptides. Theoretical MS/MS spectra could be stored in a suitable peptide database (called target database), built starting from a set of known proteins annotated in a reliable public database, such as Swiss-Prot [10].

The comparison between detected and theoretical MS/MS spectra can be carried out through a similarity scoring function and the peptides in the target database are ranked according to their scores. The experimental MS/MS spectrum is finally associated with the peptide with the highest score.

A less rough procedure involves an alternative step in which the statistical significance of each peptide in the ranked list is assessed [11–19]. The most common approach to the evaluation of statistical significance is based on the parallel analysis of a random sequence database (called decoy database), created by reversing or shuffling each amino acid sequence in the target database. The experimental MS/MS spectra are compared to the peptides in both the target and decoy databases. Two separated ranked lists are produced, according to the obtained scores. The decoy list is used to assign to each score of the target database the probability that the score is not different from those of decoy peptides and then to define a threshold that separates high and low score values.

Although unmodified peptides are automatically and efficiently identified by consolidated algorithms (e.g., ProteinProspector [20, 21], Mascot [22, 23], Sequest [24], X! Tandem [25], and OMSSA [26]), phosphopeptide MS/MS spectra often require a manual interpretation. This step represents the main bottleneck of the analysis in terms of time, because MS/MS technique generates a large amount of data, and in terms of reproducibility, since the obtained results have undergone a subjective evaluation.

To make the phosphopeptide analysis more objective, specific algorithms have been proposed in the literature for both phosphopeptide identification and phosphosite localization [27–30]. Specific drawbacks of such tools are that some of them implement postprocessing algorithms which rely on prior peptide identification via, for example, Sequest or Mascot [27, 28, 30], thus not providing a fully integrated analysis workflow; the closed source implementation of some software tools prevents the manipulation of the algorithms for optimization purposes [28]; neutral loss peaks, which are an important feature of phosphopeptides (see Section 2 for details), are not always taken into account [28]. In this work, a Perl/C procedure called PhosphoHunter is proposed, which specifically covers all the steps of phosphopeptide identification in a fully automated fashion. The tool is available on the http://aimed11.unipv.it/PhosphoHunter website for free download for nonprofit institutions.

PhosphoHunter is able to create the database of phosphorylated peptides from a set of proteins of interest, discard the MS/MS spectra that probably do not correspond to phosphopeptides, identify the remaining spectra, and localize the phosphorylated amino acids via a statistics-based procedure. It has been tested on a dataset of 1500 publicly available MS/MS spectra obtained from human peptides [31–35] and the performance of PhosphoHunter has been evaluated via comparison with two other software tools: Mascot [22, 23] (a widely used tool, which is not specific for phosphopeptides but is used as a benchmark in other works describing phosphopeptide identification methods [27, 28]) and Inspect [29] (a phosphopeptide-specific tool).

## 2. Methods

**Figure 1:**
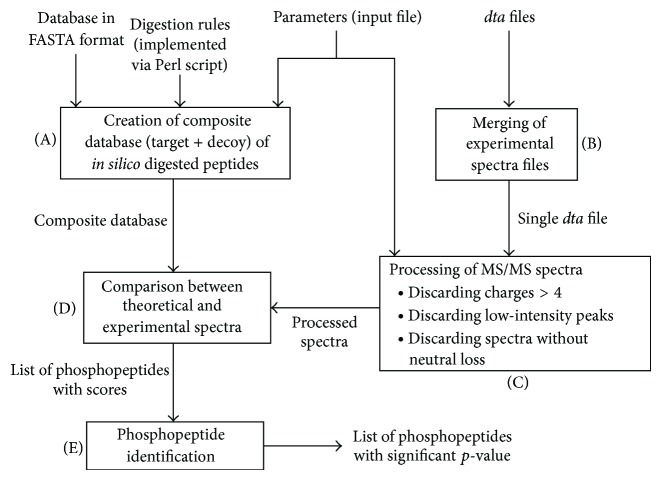
Summary of PhosphoHunter procedure. Block A (implemented via the* create_database.pl* script): a database in FASTA format is used to create a target database according to appropriate digestion rules and other parameters provided in an input file, such as the number of allowed consecutive missing cleavages. The database is then used to obtain a decoy database and a single composite database is obtained from target and decoy databases. Block B (implemented via the* merge.pl* script): individual* dta* files, corresponding to experimental spectra, are merged into a single* dta* file. Block C: experimental spectra are normalized and processed by discarding charges higher than 4, low-intensity peaks, and peptides not showing neutral loss. The intensity threshold of neutral loss is specified in the input file. Block D: theoretical and processed spectra are compared according to a scoring function and a list of phosphopeptides with scores is associated with each spectrum. Block E: for each spectrum, a *p*-value is computed for each element of the list and only the peptides with a *p*-value below a specific threshold, defined in the input file (relation not shown in the figure), are kept in the final list. Blocks C, D, and E are all implemented via the* phosphopeptide_ID.pl* script.

PhosphoHunter includes the following four main steps (see Figure 1):

1. creation of a database of theoretical peptides by* in silico* digestion of a set of proteins;
2. processing of the MS/MS experimental spectra;
3. comparison between the processed MS/MS spectra and theoretical peptides;
4. phosphopeptides identification.

### 2.1. Database Creation

The database of peptides is created by following the method proposed in [36]. Starting from a list of FASTA format protein sequences coming from a database such as Swiss-Prot, PhosphoHunter carries out the* in silico* digestion of the amino acid sequences to obtain the theoretical peptides. The* in silico* digestion follows the specific rules of the protease actually used during the preparation of the biological sample. In particular, the* create_database.pl* script contains the regular expressions corresponding to the trypsin digestion rules [37]. Trypsin is the only implemented protease in the current version of the tool, although any other protease may be considered, since new sets of appropriate rules can be added by users via Perl script. Specifically, the* create_database.pl* has to be modified where the* #enzymatic digestion* comment is reported and rules have to be overwritten to perform an* in silico* digestion with the desired protease. As an example of that, PhosphoHunter distribution includes an additional file (*create_database_chymo.pl*), where it is shown how to perform the digestion with a different protease (i.e., chymotrypsin), whose digestion rules are reported in [37] (low specificity version).

The target database may be customized in terms of investigated organism(s), missed cleavages (MCs), and PTMs, depending on the expected characteristics of the specific study. For each protein, the theoretical mass of every peptide is computed as described in [36]. The whole set of mass values is the theoretical MS spectrum of the protein (also called theoretical peak list). In the searching step (step #3), the peak list will be used to compare the peptides in the target database with the ones in the experimental spectra, to find the most similar ones.

To test the statistical significance of the results, a decoy database is built [11–19] by reversing all the protein sequences of the target database and performing the same* in silico* digestion procedure. The two databases (i.e., target and decoy) are merged into a single database, hereafter called composite database.

### 2.2. MS/MS Data Processing

First, all the acquired MS/MS spectra have to be converted from raw to* dta* files using the desired preprocessing algorithm, such as the ones described in [8, 9]. Each file contains the following data referred to a single MS/MS spectrum: (i) the* m/z* value of the precursor, (ii) the charge of the precursor, and (iii) the list of* m/z* and intensity values of all the detected fragment ions. All the* dta* files are merged automatically into a single ASCII file, by using the Perl script* merge.pl*, included in PhosphoHunter distribution. The next subsections describe the strategy applied to process the resulting ASCII file and to select the MS/MS spectra that are likely to correspond to phosphorylated peptides.

#### 2.2.1. Discarding the Highest Charge States

The precursors (and the related MS/MS spectra) with a charge greater than four are removed from the ASCII file. In fact, the interpretation of their MS/MS spectra is typically not reliable because of the low efficiency of the CID fragmentation [38].

#### 2.2.2. Discarding the Least Intense MS/MS Signals

Given a precursor, the intensities of its fragment ions are normalized to the most intense peak. Its normalized intensity is 100. The fragment ions are sorted by* m/z* and the* m/z* values are split in bins, each containing one hundred peaks. The fifty less intense peaks of each bin are filtered out, removing a large part of the noise. In all the steps described below, the normalized intensity of the peaks in experimental spectra will be considered.

#### 2.2.3. Neutral Loss Analysis

Phosphorylated peptides with phosphoserine or phosphothreonine show a typical MS/MS spectrum often dominated by an intense peak, which corresponds to a neutral loss of H_3_PO_4_ [38, 39]. Neutral loss is a particular behaviour showed by a phosphopeptide during CID fragmentation. It is due to weakness of the chemical bond of the phosphate group, which has less energy than others and competes with the backbone bonds during the fragmentation of the peptide. Exploiting this feature, the potential phosphopeptide MS/MS spectra can be qualitatively separated from the other MS/MS spectra, reducing the overall complexity of the analysis. For each experimental spectrum, the potential* m/z* values where a neutral loss peak can be observed are derived by knowing the precursor mass. Only the spectra showing peaks in this position (within a tolerance) with an intensity over a given threshold are selected; these peaks should correspond to the neutral loss of H_3_PO_4_. The intensity threshold used by PhosphoHunter to select the MS/MS spectra is a tunable parameter chosen by the user. Importantly, the threshold intensity has to be chosen according to the investigation aims, to minimize the false discovery rate, to increase the accuracy, or to optimize other parameters of interest. The selected MS/MS spectra are then compared to the theoretical spectra in the composite database, generated in step #1, to identify the corresponding phosphopeptide sequences and to assess the statistical significance of the matching.

### 2.3. Searching Step

To determine the most likely amino acid sequence corresponding to an experimental MS/MS spectrum, two comparisons are performed. First, the precursor mass value is compared to the theoretical mass of the peptides in the composite database, using an absolute mass tolerance in dalton (Da) or a relative mass tolerance in parts-per-million (ppm), as specified by the user in the input file. A match between the theoretical mass *y*_*i*_ and the precursor mass *x*_*j*_ occurs if |*x*_*j*_ − *y*_*i*_ | ≤*δ*_*j*_, where *δ*_*j*_ is the mass tolerance fixed for the precursor mass *x*_*j*_. If this match occurs, a second comparison is performed: the MS/MS spectrum generated by the fragment ions of the precursor is compared to the theoretical spectrum built from the matching peptide sequences. This second comparison allows to determine the amino acid sequence of the peptide generating the acquired MS/MS spectrum and the phosphorylation sites along the peptide itself. To better understand how the proposed procedure generates the theoretical MS/MS spectrum given a peptide, a detailed description of the fragmentation process is here provided. During the second stage of the MS/MS, peptides are fragmented along the backbone [38]. The mass spectrometer detects only the fragment ions that retain the precursor charge(s), whereas the neutral fragments are invisible. Depending on the collision energy, different ion species can be present. Low energy collision, like those used in CID (e.g., 25–70 eV), are usually preferred because higher values of energy produce many fragment ion types, making the interpretation of the MS/MS spectrum harder. The ions produced by CID are mainly of b- and y- ion series. The fragment ions may lose a water molecule (H_2_O, −18.011 Da) or an ammonia molecule (NH_3_, −17.027 Da) during the fragmentation process, producing further types of detected masses (*m/z*) in the MS/MS spectrum. The fragmentation model implemented in PhosphoHunter to generate theoretical spectra takes into account the b- and y-ion series and the neutral loss of water, of ammonia and of H_3_PO_4_. The spectra comparison strategy is herein described. Let us consider a peptide in the database. Given its amino acid sequence, we immediately know the total number of possible phosphorylation sites, corresponding to all the serine, threonine, and tyrosine. If the total number of phosphorylated sites of this peptide is also known, two cases may occur: either the number of possible phosphorylation sites is equal to the number of actually modified amino acids or it is greater. In the former case, there is not uncertainty on the localization of the phosphorylation sites and then the only possible MS/MS spectrum is generated from the peptide sequence. For example, if the peptide SCPEDCK has a phosphorylated site, since it has only one serine and no threonine or tyrosine, only the MS/MS spectrum of the sCPEDCK is created (where the modified serine is represented by a lower case “s”). Conversely, if the localization of phosphorylation sites is uncertain a theoretical spectrum for each possible situation is created. For example, considering the peptide FSAASSASK (with four serine residues) and assuming that the precursor mass allows us to infer that two amino acids are phosphorylated, the conceived procedure generates the theoretical spectrum for the following peptides: FsAAsSASK, FsAASsASK, FsAASSAsK, FSAAssASK, FSAAsSAsK, and FSAASsAsK. All the resulting theoretical MS/MS spectra are then compared with the experimental one by using a scoring function to correctly localize the phosphorylation sites. As before, the comparison is performed using a mass tolerance (in Da or in ppm, specified by the user in the input file). The scoring function implemented in PhosphoHunter ranks the peptides in the composite database according to the (weighted) number of matches between experimental and theoretical MS/MS spectra. More precisely, let us consider a peptide in the database whose theoretical MS/MS spectrum matches *M* peaks (the fragment ions) of the experimental MS/MS spectrum within the given mass tolerance. The comparison between spectra is performed by scoring each matching peak by the following sigmoid function:

(1) $$\begin{array}{c} y\left(I_{j}^{E}\right)=\frac{2}{1+e^{-I_{j}^{E}}}-1, \end{array}$$

where *I*_*j*_^*E*^ is the intensity of the *j*th experimental peak that matches the theoretical MS/MS spectrum. This score assigns a value between 0 and 1 to each matching peak. The total peptide score is then computed as

(2) $$\begin{array}{c} \text{Score}=\overset{M}{\underset{j=1}{\sum }}y\left(I_{j}^{E}\right)=\overset{M}{\underset{j=1}{\sum }}\left(\frac{2}{1+e^{-I_{j}^{E}}}-1\right). \end{array}$$

The use of the sigmoid function defined above enables the weighting of peaks according to their intensity. In fact, low-intensity peaks may represent actual signal or may be caused by noise. For this reason, such peaks are considered more uncertain than high-intensity ones and their weight in the scoring function is lower. A simple discrete function assigning 1 to matched peaks would not have enabled the intensity-dependent weighting of peaks.

### 2.4. Phosphopeptide Identification

Peptides whose theoretical spectrum matches at least one peak of the experimental MS/MS spectrum (here called hits) are ranked through the scoring function (2). In a statistical framework, it is possible to find a score threshold (called critical value) able to reject, with a desired degree of certainty, the null hypothesis that the score of a hit is not greater than the ones obtained by chance. To this aim, the searching step (step #3) is performed in the composite database (target and decoy) and two separated and ranked hit lists of true and random peptides, sorted by decreasing score, are generated.

Since the statistical significance of the score of each hit in the target list has to be assessed, the number of actually performed statistical tests is very large [14]. To control the Type I error of the whole procedure, that is, to avoid a frequent rejection of the null hypothesis when it is true, multiple statistical test corrections have to be used. They are based on the idea that if *K* null hypotheses have to be tested, the critical value *α* of each single test needs to be lowered to account for the number of comparisons being performed. Among all the possible criteria, the Bonferroni correction is here adopted. In this case, *α* becomes *α*_whole_/*K*, where *K* is the number of target peptides in the composite database that are tested as null hypotheses. This value can be used to determine the score threshold from the decoy list. Alternatively, the distribution of the decoy hit scores of each spectrum can be used to compute, for each hit of the target list, *P*  (*S* ≥ *s*); that is, the probability that a score *S* greater than the one of the considered hit (indicated with *s*) can be obtained if the hit belongs to the decoy database (null hypothesis of the test). This probability is the *p*-value associated with the hit and if it is lower than *α* it can be used to reject the null hypothesis that the target peptide randomly matches the experimental spectrum.

## 3. Results and Discussion

A dataset of consensus MS/MS spectra was analyzed in order to assess the performance of PhosphoHunter. Phosphopeptide identifications were compared with those obtained using Mascot and Inspect. PhosphoHunter ran on one node of a computer cluster with the Linux SUSE Enterprise 9.3 distribution. The node had a Quad-Core Intel Xeon processor X5355 with a 8 GB RAM.

### 3.1. Dataset

A test dataset of 1500 MS/MS spectra was used, selected in accordance with the criteria reported below, from two publicly available spectral libraries of human peptides (i.e., the Institute for Systems Biology (ISB) and the National Institute of Standards and Technology (NIST) libraries of peptides [31–35]).

- The ISB dataset was created by Bodenmiller and colleagues [31, 32]. They identified the MS/MS spectra using Sequest and the phosphorylation sites were then validated using the PeptideProphet software tool [40, 41]. Peptides, identified from highly phosphopeptide-enriched protein samples, are both tryptic and semitryptic. They are characterized, in terms of PTMs, by the phosphorylation of serine, threonine, and tyrosine as variable modification and carboxyamidomethylation of cysteine as fixed modification. The charge state of the precursors is two (3336 MS/MS spectra) or three (1757 MS/MS spectra). The whole dataset contains 5093 MS/MS spectra, 4193 of which correspond to phosphopeptides.
- The NIST library contains 12473 MS/MS spectra. Peptides are tryptic and nontryptic, as above; the considered PTMs are the carboxyamidomethylation of cysteine, the oxidation of methionine, and the acetylation of several amino acids. The charge state of the precursors ranges from one to five (involving 55, 7973, 3817, 627, and 1 MS/MS spectra, resp.). There are no phosphorylated peptides.

Both datasets report the probability of correct identification of the amino-acid sequence for each peptide. Starting from these two sources, a new well annotated test dataset containing phosphorylated tryptic peptides was built in accordance with the following criteria.

1. The MS/MS spectra corresponding to peptides containing the letters B, X, or Z (B, X, and Z are codes associated with ambiguous amino acids: B is aspartic acid or asparagine, X is an unknown amino acid, and Z is glutamine or glutamic acid) were discarded to avoid the presence of sequences with unknown amino acids.
2. The carboxyamidomethylation of cysteine (fixed) and the phosphorylation of serine and threonine (variable) were the only considered PTMs. Peptides in which amino acids were modified by other PTMs were removed. In this context, the phosphorylation of tyrosine was not considered, since it hardly causes neutral loss peaks under CID conditions.
3. Only tryptic peptides were considered.
4. The MS/MS spectra acquired from precursors with charge states greater than four were not considered for the reasons discussed in Section 2.
5. The remaining MS/MS spectra were selected on the basis of the probability of correct identification, annotated in the source databases, taking into consideration only the MS/MS spectra with probability greater than 0.99. This very high probability threshold allowed us to have a high confidence in the identity of the peptide when the performance of PhosphoHunter was tested and compared with Mascot and Inspect.
6. At the end of the spectrum selection procedure, 750 phosphorylated peptides were considered from the ISB dataset and 750 MS/MS spectra were also selected from the NIST dataset to have a balanced test set (the complete list of spectra considered in this work is available at http://aimed11.unipv.it/PhosphoHunter/Spectra.rar).

Therefore, the test dataset contained 1500 MS/MS spectra. The charge states of the precursors were two, three, and four (involving 1141, 332, and 27 MS/MS spectra, resp.).

### 3.2. Composite Database

A target database was created as reported in [36]. The database contained* Homo sapiens* tryptic peptides, generated starting from the 57.12 release of Swiss-Prot (20,287 human amino acid sequences). A maximum of two consecutive MCs were allowed for each tryptic peptide (this parameter is set in the tool input file). The PTMs considered were the phosphorylation of serine and threonine (+79.97 Da) and the carboxyamidomethylation of cysteine (+57.02 Da). The final number of target peptides in the database was about 10^7^. This value was used to correct the critical value *α* in the identification step, when the statistical significance of the identification results was assessed [42]. The decoy database was generated starting from the same* Homo sapiens* proteins, by reversing their sequences to obtain 20,287 new random proteins [12]. These proteins were appended to the target database, creating the composite one containing 40,574 proteins.

### 3.3. Phosphopeptide Identification

First, MS/MS spectra were processed as reported in step #2 (see Section 2) to prepare suitable files and to analyze only the spectra that show a significant neutral loss signal, typical of phosphopeptides. Neutral loss analysis was performed by tuning the intensity threshold from 0 to 100 and by using a tolerance of 3 Da divided by precursor charge. The results, in terms of number of selected spectra, are reported in Table 1, in which eleven intensity values were considered. Intuitively, the number of the selected spectra decreases when the intensity threshold increases. It is worth noting that, even when the threshold is set to 0, some actually nonphosphorylated spectra are nevertheless discarded, because only the MS/MS spectra having one or more specific neutral loss signals of any intensity were selected (in this case, 488 over 750); on the other hand, all the spectra corresponding to actually phosphorylated peptides were selected. Increasing the threshold from 0 to 100, the number of nonphosphorylated MS/MS spectra that passes the check decreases (from 488 to 11), but this also occurs for the phosphorylated peptides that decrease from 750 to 462. The neutral loss analysis is highly specific, as shown by the percentage of phosphorylated peptides that pass the test, which increases monotonically with threshold increasing (from 61% without selection to 98% with the highest threshold value). The benefit of using neutral loss analysis is twofold: lowering the computational time required for the analysis of the whole dataset (only the most promising candidates are considered) and limiting the presence of wrong identifications because a significant part of nonphosphorylated peptides is filtered out. Then, the MS/MS spectra that passed the neutral loss check were searched in the composite database (step #3) and the target and decoy ranked lists were generated for each spectrum. The mass tolerance used was 7 ppm for the precursor ion masses and 0.8 Da for the fragment ions. The *p*-values were computed for all the target hits and the ones with a *p*-value smaller than *α* were considered as statistically significant. The *α*_whole_ value was set to 0.05 and it was corrected for multiple testing, using the number of peptides in the target database. Therefore, *α* was set to 5 · 10^−9^.

Using the hit lists, two analyses were conducted to answer the two typical questions addressed in this kind of study: that is, are phosphorylated peptides correctly recognized? Are phosphorylation sites correctly recognized?

The first question can be addressed by PhosphoHunter in two ways: considering as a phosphorylated peptide each corresponding spectrum that passes the neutral loss check and has either at least one entry in the target hit list or at least one significant entry in the target hit list. The results of this analysis, performed on the test dataset, are summarized in Tables 2 and 3, respectively. Both tables illustrate the performance of PhosphoHunter in terms of true positives, false positives, true negatives, false negatives, sensitivity, specificity, overall accuracy, false discovery rate (FDR), precision (already reported as percentage in Table 1), and *F*-measure [43, 44]. By increasing the intensity threshold from 0 to 100, the number of false positives decreases from 488 to 11 and from 240 to 3 for all the hits and the significant ones, respectively. Consequently, the specificity increases. On the other hand, unfortunately, the number of false negatives increases as well and thus the sensitivity decreases. Focusing on FDR as a measure of PhosphoHunter performance, it achieved values of about 0.05 when the intensity threshold was set to 60 and 40 (the latter when statistical validation was considered). Although the choice of preferring a high specificity or sensitivity depends on the particular problem under investigation, both the overall accuracy and *F*-measure, which make a compromise between the two different choices, suggest, for this study, an optimal threshold of 20–30. However, the neutral loss intensity threshold remains a tunable parameter for the analyst and it has to be set in accordance with his/her preferences. Interestingly, according to the computed indexes reported in Tables 2 and 3, it is to remark that the performances of PhosphoHunter in presence of the statistical validation are better than those without this validation. In particular, FDR, precision, recall, *F*-measure, accuracy, and specificity are systematically better with statistical validation than without validation, while, on the other hand, sensitivity becomes lower due to the restrictive contribution of *p*-value. In conclusion, Tables 2 and 3 demonstrate the usefulness of both the neutral loss check and the statistical validation of the results.

The second question concerned the ability to correctly identify the phosphorylated peptide sequences and even the phosphorylation sites along the peptide with a certain degree of confidence. PhosphoHunter generates a ranked scored list of hits at the end of its workflow. The desirable situation is to obtain a very short list (ideally only one hit) in which the true peptide is present in the first position with a score significantly higher than other ones. For example, let FsAASSASK be the amino acid sequence corresponding to one MS/MS spectrum in which the first serine is phosphorylated. Through the identification step, PhosphoHunter includes several sequences in the candidates list. If both the FsAASSASK and FSAAsSASK peptides are included, it is clear that both of them have the correct amino acid sequence, but the phosphorylation site is correctly identified only in the first one. For each spectrum we verified if the right sequence and the right phosphorylation site were included in the hit list, considering eleven neutral loss thresholds. Results are summarized in Table 4 both considering all the hits and only the statistically significant ones. The sequences of all the spectra that pass the neutral loss check are correctly identified. Among these sequences, about 91% have correctly detected phosphorylation sites, independently from the used threshold and statistical validation. Although the illustrated results with and without statistical validation appear to be similar, as already observed above, the number of false positives is significantly reduced after the statistical check and the ranked hit list is significantly shortened also for the true positive spectra, with evident benefits for the analyst (results not shown).

#### 3.3.1. Comparison with Mascot

The comparison between PhosphoHunter and Mascot was performed on the same test dataset using Mascot Daemon release 1.2. The same searching parameters of PhosphoHunter were set in Mascot. It is important to remark that the comparison is not trivial because the performance of PhosphoHunter depends on the intensity threshold used to filter the MS/MS spectra before the identification, whereas Mascot does not include a routine for nonphosphorylated peptides filtering, and then it tries to identify all the submitted MS/MS spectra. However, it includes a method to test the statistical significance of the hit scores [22, 23]. Therefore, in the following analyses only statistically significant scores were considered. Both the ability of recognizing phosphorylated peptides and true peptide sequences were assessed on the spectra of the test dataset. Results are summarized in Tables 5 and 6, respectively. From Table 5, it is possible to see that Mascot correctly recognized all the nonphosphorylated spectra but wrongly classified more than 25% of the phosphorylated spectra. This can constitute an important weak point if the analysis is focused on the discovery of phosphorylated peptides. It is not trivial to increase sensitivity (decreasing specificity), due to the lack of a tunable parameter, which is present in the PhosphoHunter tool. In addition, global performance indexes such as accuracy and *F*-measure are worse than the ones obtained with PhosphoHunter (compare Table 3 with Table 5), using a reasonable threshold of 10–30 as a term of comparison. Results were not compared with the ones corresponding to the optimal neutral loss threshold of Table 1 to avoid overfitting problems and, consequently, optimistic polarized conclusions.

The performance on the identification of correct phosphorylated sites gave similar results to PhosphoHunter (see Table 6): Mascot identified all the correct sequences and correctly recognized 93% of the sites. In conclusion, the comparison with Mascot highlighted the very good performance and the flexibility of the ad hoc developed procedure implemented in PhosphoHunter to identify phosphorylated peptides from MS/MS spectra.

#### 3.3.2. Comparison with Inspect

The comparison between PhosphoHunter and Inspect was performed using the stand-alone version of the 2009 release and its enclosed Python scripts, downloaded from the http://peptide.ucsd.edu/ website, using the same searching parameters as above. Similarly to PhosphoHunter, Inspect implements a full analysis workflow, carried out via a different procedure. As recommended in the Inspect tutorial (Sam Payne, winter 2007), the decoy database was created via the* ShuffleDB.py* routine with default parameters, it was appended to the target database, and the resulting composite database was converted into the* trie* format. After the Inspect run, the* PValue.py* routine was used with the empirical method, as recommended, to select only the statistically significant scores. As in the case of Mascot, the comparison with PhosphoHunter is not trivial, since it depends on the neutral loss intensity threshold. Results are summarized in Tables 5 and 6, respectively. Table 5 shows that Inspect correctly recognized the majority of nonphosphorylated spectra, thus resulting in a higher specificity, precision, and lower FDR value than PhosphoHunter. This result is similar to the one obtained with Mascot. However, by making a comparison with PhosphoHunter at the same threshold as above (10–30), Inspect had a lower sensitivity (87%), although it is higher than the one of Mascot tested on this dataset. For this reason, in the illustrated conditions PhosphoHunter is expected to find more phosphorylated spectra than the other two tools and it also includes a parameter (intensity threshold) to intuitively tune sensitivity and specificity according to user needs. On the other hand, in these conditions, Inspect has slightly better values than PhosphoHunter in terms of FDR, precision, *F*-measure, and overall accuracy. These indexes can change according to the PhosphoHunter intensity threshold, as described above (see Table 3 for a threshold-dependent performance index list). For example, if specificity has to be maximized, the threshold can be increased to reach a value comparable to the one of Inspect. For each index, PhosphoHunter can reach (and possibly outperform) Inspect performances. Finally, as reported in Table 6, Inspect correctly identified 99% of the true positive sequences and, among them, it correctly localized 95% of the phosphosites.

Examples of experimental spectra where PhosphoHunter was successful and Inspect and/or Mascot were not are herein provided, considering, for instance, a threshold of 30 and statistical validation for PhosphoHunter. The TPVsPVK phosphopeptide (spectrum with the 1054_1054 code in the available dataset) was successfully identified by PhosphoHunter, even by increasing the neutral loss threshold to 100, but it was not correctly classified by either Inspect or Mascot, where no significant entries were present in the hit list. The LLPSAPQTLPDGPLAsPAR phosphopeptide (code 1343_1343) was correctly identified by PhosphoHunter and Mascot, but Inspect classified it as a nonphosphorylated peptide with wrong sequence. On the other hand, the ANtPELK phosphopeptide (code 1058_1058) was correctly classified by PhosphoHunter and Inspect, but not by Mascot, where no significant entries were present in the hit list. Considering phosphosite localization, the TAsGSSVTSLDGTR phosphopeptide (code 1459_1459) was correctly classified by PhosphoHunter, while both Inspect and Mascot failed in the phosphosite identification and provided the same wrong sequence (tASGSSVTSLDGTR) as significant entry in their hit list, although they successfully classified the spectrum as a phosphopeptide and correctly identified the amino acid sequence.

## 4. Conclusions

In this paper, a new Perl software tool, called PhosphoHunter, is proposed for the analysis of phosphopeptide MS/MS spectra and it was tested on a dataset of 1500 MS/MS consensus spectra. While many available software tools implement phosphopeptide-specific postprocessing algorithms, downstream of consolidated peptide search tools, PhosphoHunter provides a full and automated analysis pipeline, starting from* dta* files (and a FASTA format database) and returning phosphopeptide identification and phosphosite localization. Importantly, if the analysis is focused on a specific subset of a proteome (e.g., the human amniotic fluid or the plasma proteome), PhosphoHunter allows us to create a database from a pool of selected proteins, reducing the number of false positive identifications and speeding up the analysis. Moreover, it includes a preliminary step to filter the MS/MS spectra that do not have the typical signals indicating the presence of some phosphorylated amino acids. Another advantage of PhosphoHunter is that it is implemented in Perl/C and the user can easily extend and customize its features. Many parameters can be set directly in the input file, as indicated above, while others (e.g., the peptide cleavage rules) can be easily changed via Perl script modifications. On the other hand, as it was anticipated in the Background section, the purpose of the proposed procedure is not only the identification of phosphorylated peptides, but also the increase of analysis efficiency and reproducibility. In fact, the inspection of MS/MS spectra by expert users is time consuming, because of the large amount of data generated by the mass spectrometer and of the complexity of the signals acquired. The filtering step (step #2) allows us to decrease the number of MS/MS spectra that the procedure has to identify, by the detection of particular phosphorylation signals in the MS/MS spectrum. The detection of the phosphorylation peaks in the MS/MS spectrum is performed by analyzing the most intense peaks. These signals are selected using an intensity threshold defined by the analyst. The results shown in this paper highlight that the performance of PhosphoHunter (Tables 2, 3, and 4) depends on this threshold because it determines the number of phosphopeptides that the procedure analyzes and identifies. The threshold is a parameter of the software tool and it has to be chosen by the user in accordance with the specific investigation to perform (e.g., to increase accuracy or to reduce FDR). Another feature that makes the procedure robust is the statistical validation of the results. When a phosphopeptide is identified, a ranked list of all the possible candidate peptides is produced by PhosphoHunter. This list contains true and random peptides. For each hit in that list, a *p*-value, indicating the probability of the candidate of being identified by chance, is computed through multiple hypothesis tests. As multiple tests are performed, the critical value, *α*, needs to be lowered to account for the number of comparisons being performed. The number of comparisons is equal to the number of target peptides in the composite database. The statistical validation allows the reduction of the number of candidate hits for each phosphopeptide, because the candidates achieving a *p*-value greater than the threshold *α* chosen by the user (usually *α* = 0.05/*K*, where *K* is the number of target peptides in the composite database) are random hits by definition. It is also important to highlight that the implemented tool is affected by two specific limitations. Since its filtering step is based on neutral loss peak recognition, only CID data can be analyzed, while other fragmentation methods, such as the electron transfer dissociation (ETD) method, do not cause neutral losses. Moreover, since phosphotyrosine hardly shows neutral loss under CID conditions [45], peptides containing one or more phosphotyrosine residues and no phosphoserine or phosphothreonine (which give neutral loss signals) are not selected and the phosphorylation of tyrosine is not taken into account as a variable modification in the following steps of the algorithm. Concerning these limitations, it is important to highlight that CID is still commonly used as peptide fragmentation method, while tyrosine phosphorylation occurs in low percentage compared to serine and threonine [31, 45–48]. Other software tools and studies also neglect phosphotyrosine in their analyses [30, 45]. For these reasons, the application field of PhosphoHunter is very wide. PhosphoHunter (with statistical significance, at a threshold of 10–30) was compared with Mascot, a very popular software tool. Results showed that the overall performance of PhosphoHunter was better than Mascot in terms of sensitivity, accuracy, and *F*-measure. Mascot gave no false positives, inherently resulting in better specificity and FDR and precision values than PhosphoHunter. However, it is important that if the study aims at identifying phosphopeptides, Mascot wrongly classifies more than 25% of the spectra, as opposed to PhosphoHunter, which is characterized by a much higher and tunable sensitivity. PhosphoHunter was also compared with the Inspect tool to provide a comparison with a full phosphopeptide identification workflow. The overall performance of Inspect was comparable with PhosphoHunter, considering a threshold of 10–30: whereas Inspect performed slightly better in terms of accuracy, FDR, *F*-measure, precision, and specificity, PhosphoHunter had a higher sensitivity, which reflects a high capability of discovering phosphorylated spectra. As already anticipated in Section 3, many PhosphoHunter results are threshold-dependent. For this reason, although an intensity threshold of 10–30 was considered as an example in the discussion of performance results, the final user should consider the full list of performance index values, reported in Tables 1, 2, and 3. This can guide the tuning of the threshold value to maximize sensitivity, specificity, or other indexes, according to user needs. It is worth noting that the development of PhosphoHunter did not require the tuning of several parameters by evaluating the algorithm on a training and a test set, but the tool was expressly designed including a single, intuitive, performance-related parameter (i.e., the intensity threshold of neutral loss) that users can freely fix and tune according to the aim of their analysis. In other words, we did not search for an optimal threshold parameter, since our aim is to provide a flexible procedure allowing sensitivity and specificity to be tuned according to the user's preference. For the reasons above, the analysis of the dataset considered in this study was not finalized to the learning of an optimal threshold value for future use and the value showing the highest accuracy and *F*-measure on this dataset (20–30) should not be considered as a recommended threshold, since it may change according to the analyzed dataset. The above threshold values were only used in this study to compare the performances that PhosphoHunter can reach with the ones of two other tools. Apart from the neutral loss intensity threshold, the other parameters of the proposed procedure, such as the number of allowed consecutive missing cleavages during* in silico* digestion and *p*-value threshold, are the same usually required by other software tools. Commonly adopted values, also suggested by these tools, are reported in the default input file of PhosphoHunter distribution. On the other hand, some parameters depend on experimental data, such as mass and fragment tolerance, and then they should be set according to the specific MS/MS equipment used in the analysis.

PhosphoHunter, Mascot, and Inspect were all characterized by a high recognition capability of peptide sequences and phosphosites, with 100%, 100%, and 99% of correctly identified sequences (present in the hit list) among the true positive spectra and 91%, 93%, and 95% of correct phosphosite localization in these sequences, respectively.

In summary, this study and the two performed comparisons with other tools highlighted that PhosphoHunter performances are comparable to Inspect, another phosphorylation-specific tool, while both of them were found to be superior to Mascot, a widely used but phosphorylation-unspecific tool. The procedure implemented in PhosphoHunter is highly flexible according to user needs and it can be easily customized by tuning a single performance-related parameter. The procedure is also relatively simple and intuitive and it formalizes the manual interpretation process of MS analysis experts. PhosphoHunter has been used for phosphopeptide identification in a recent publication [49] and is currently in use at the Dipartimento di Scienze del Farmaco (Università degli Studi di Pavia).

## Figures and Tables

**Table 1:** Number of MS/MS spectra selected through the analysis of the neutral loss peaks.

Intensity threshold	Selected MS/MS spectra			
	Phosphorylated	Nonphosphorylated	Total	%
0	750	488	1238	60.6
10	727	239	966	75.3
20	703	147	850	82.7
30	664	98	762	87.1
40	634	70	704	90.1
50	603	49	652	92.5
60	579	34	613	94.5
70	548	24	572	95.8
80	520	19	539	96.5
90	493	14	507	97.2
100	464	11	475	97.7

Intensity threshold values (from 0 to 100) and number of MS/MS spectra which pass the neutral loss check, subdivided by phosphorylated and nonphosphorylated peptides. Percent of phosphorylated peptides over the total number of selected spectra.

**Table 2:** Performance of PhosphoHunter in terms of phosphorylated spectra detection (1500 MS/MS spectra analyzed: 750 phosphorylated peptides + 750 nonphosphorylated peptides) without statistical validation.

Intensity threshold	TP	FP	FN	TN	Sens	Spec	Acc	FDR	Prec	*F*
0	750	488	0	262	1.000	0.349	0.675	0.394	0.606	0.755
10	727	239	23	511	0.969	0.681	0.825	0.247	0.753	0.847
20	703	147	47	603	0.937	0.804	0.871	0.173	0.827	0.879
30	664	98	86	652	0.885	0.869	0.877	0.129	0.871	0.878
40	634	70	116	680	0.845	0.907	0.876	0.099	0.901	0.872
50	603	49	147	701	0.804	0.935	0.869	0.075	0.925	0.860
60	579	34	171	716	0.772	0.955	0.863	0.055	0.945	0.850
70	548	24	202	726	0.731	0.968	0.849	0.042	0.958	0.829
80	520	19	230	731	0.693	0.975	0.834	0.035	0.965	0.807
90	493	14	257	736	0.657	0.981	0.819	0.028	0.972	0.784
100	464	11	286	739	0.619	0.985	0.802	0.023	0.977	0.758

TP: true positive, spectra of phosphorylated peptides passing the neutral loss check and for which at least a hit was found; FP: false positive, spectra of nonphosphorylated peptides passing the neutral loss check and for which at least a hit was found; FN: false negative; TN: true negative; Sens: sensitivity or recall, TP/(TP + FN); Spec: specificity, TN/(TN + FP); Acc: accuracy, (TP + TN)/all spectra; FDR: false discovery rate, FP/(TP + FP); Prec: precision, TP/(TP + FP); *F* : *F*-measure, 2 ∗ precision ∗ recall/(precision + recall).

**Table 3:** Performance of PhosphoHunter in terms of phosphorylated spectra detection (1500 MS/MS spectra analyzed, 750 phosphorylated peptides + 750 nonphosphorylated peptides) with the statistical validation.

Intensity threshold	TP	FP	FN	TN	Sens	Spec	Acc	FDR	Prec	*F*
0	743	240	7	510	0.991	0.680	0.835	0.244	0.756	0.857
10	721	129	29	621	0.961	0.828	0.895	0.152	0.848	0.901
20	697	77	53	673	0.929	0.897	0.913	0.099	0.901	0.915
30	659	50	91	700	0.879	0.933	0.906	0.071	0.929	0.903
40	630	35	120	715	0.840	0.953	0.897	0.053	0.947	0.890
50	599	23	151	727	0.799	0.969	0.884	0.037	0.963	0.873
60	575	18	175	732	0.767	0.976	0.871	0.030	0.970	0.856
70	544	12	206	738	0.725	0.984	0.855	0.022	0.978	0.833
80	516	9	234	741	0.688	0.988	0.838	0.017	0.983	0.809
90	489	6	261	744	0.652	0.992	0.822	0.012	0.988	0.786
100	460	3	290	747	0.613	0.996	0.805	0.006	0.994	0.758

TP: true positive, spectra of phosphorylated peptides passing the neutral loss check and for which at least a hit was found; FP: false positive, spectra of nonphosphorylated peptides passing the neutral loss check and for which at least a hit was found; FN, false negative; TN: true negative; Sens: sensitivity or recall, TP/(TP + FN); Spec: specificity, TN/(TN + FP); Acc: accuracy, (TP + TN)/all spectra; FDR: false discovery rate, FP/(TP + FP); Prec: precision, TP/(TP + FP); *F* : *F*-measure, 2 ∗ precision ∗ recall/(precision + recall).

**Table 4:** Performance of PhosphoHunter in terms of sequence detection and phosphorylation sites localization.

Intensity threshold	Sequences identified		Sequences identified	
	(all the hits)		(hits with *p*-value ≤ *α*)	
	Correct sequences	Correct sites	Correct sequences	Correct sites
0	750	678	743	671
10	727	658	721	652
20	703	636	697	630
30	664	598	659	593
40	634	572	630	568
50	603	544	599	540
60	579	523	575	519
70	548	499	544	495
80	520	477	516	473
90	493	452	489	448
100	464	425	460	421

The 750 MS/MS spectra of phosphorylated peptides were considered and the intensity threshold was tuned from 0 to 100.

**Table 5:** Performance of Mascot and Inspect in terms of phosphorylated spectra detection (1500 MS/MS spectra analyzed, 750 phosphorylated peptides + 750 nonphosphorylated peptides).

Tool	TP	FP	FN	TN	Sens	Spec	Acc	FDR	Prec	*F*
Mascot	552	0	198	750	0.736	1	0.868	0	1	0.848
Inspect	650	15	100	735	0.867	0.98	0.923	0.023	0.977	0.919

TP: true positive, spectra of phosphorylated peptides in which the first hit (or the majority) of the hit list is phosphorylated; FP: false positive, spectra of nonphosphorylated peptides in which the first hit (or the majority) of the hit list is phosphorylated; FN: false negative; TN: true negative; Sens: sensitivity or recall, TP/(TP + FN); Spec: specificity, TN/(TN + FP); Acc: accuracy, (TP + TN)/all spectra; FDR: false discovery rate, FP/(TP + FP); Prec: precision, TP/(TP + FP); *F* : *F*-measure, 2 ∗ precision ∗ recall/(precision + recall).

**Table 6:** Performance of Mascot and Inspect in terms of sequence detection and phosphorylation sites localization.

Tool	Correct sequence	Correct sites
Mascot	552	513
Inspect	646	615

The 750 MS/MS spectra of phosphorylated peptides are considered.
